# Secondary Subtalar Arthrodesis: Surgical Outcome and Predictors of Functional Outcome and Quality of Life After Bone Block Distraction vs In Situ Technique

**DOI:** 10.1177/24730114241311895

**Published:** 2025-01-24

**Authors:** Robin Eelsing, Sally Al-Sheikh, Jens A. Halm, Tim Schepers

**Affiliations:** 1 Department of Surgery, Amsterdam UMC location University of Amsterdam, Amsterdam, the Netherlands; 2 Amsterdam Movement Sciences, AMS - Musculoskeletal Health, Amsterdam, the Netherlands

**Keywords:** calcaneal, fracture, subtalar arthrodesis, complication, malunion

## Abstract

**Background:**

The outcome of a secondary subtalar arthrodesis after prior calcaneal fracture has been widely described. However, the surgical treatment has evolved significantly over the past decade, paralleling the shifts observed in primary repair strategies. Therefore, we describe the outcome following a secondary arthrodesis after an intra-articular calcaneal fracture, comparing the in situ (ISA) and bone block distraction arthrodesis (BBDA) techniques.

**Methods:**

In total, 339 patients who underwent a subtalar arthrodesis between January 1998 and November 2022 were screened for eligibility. Inclusion criteria were age ≥16 years, having undergone a subtalar arthrodesis following a calcaneal fracture, and a minimal follow-up of 1 year. Exclusion criteria were subtalar arthrodesis before January 2010 and a subtalar arthrodesis within 6 weeks of injury. A total of 259 patients did not meet the inclusion criteria, resulting in the inclusion of 80 patients with 82 fractured calcanei.

**Results:**

No significant differences between ISA and BBDA in surgical outcome were seen. Subtalar fusion was achieved in 78 of the patients (95.1%). Additionally, a deep surgical site infection occurred in 6 patients (7.8%). The American Orthopaedic Foot & Ankle Society ankle-hindfoot scale (AOFAS) and Foot Function Index (FFI) scores and the EuroQol–5 dimensions (EQ5D) index were similar between the 2 groups. However, a significantly higher EQ5D-VAS was reported by the subjects who received a BBDA (median [interquartile range], 70.0 [52.0-82.0] vs 81.0 [70.0-90.3], *P* = .021). Multiple regression revealed that a higher Böhler angle before the initial fracture reconstruction significantly improved the AOFAS score, whereas the FFI significantly improved by an initial conservative treatment and implant removal after arthrodesis. Finally, increasing age significantly improved the EQ5D index.

**Conclusion:**

Our study presents comparable surgical outcomes between ISA and BBDA for secondary subtalar arthrodesis following calcaneal fractures. Functional outcomes, as measured by the AOFAS and FFI scores, were also similar between the 2 techniques, although patients undergoing BBDA reported higher EQ5D visual analog scale scores.

**Level of Evidence:**

Level III, retrospective cohort study.

## Introduction

Subtalar arthrodesis with the intend to create fusion was first described in 1905 by Nieny^14^ for the correction of paralytic foot deformities. It was believed that an isolated fusion of the subtalar joint would negatively influence adjacent joints leading to changed motion and consequently arthritis. It was for that reason that triple arthrodesis was favored over isolated subtalar arthrodesis. Over the past 30 years, it has become commonplace to address the pathology by only inducing fusion of the affected joint.^12^

The main indication for secondary subtalar joint fusion is pain due to posttraumatic arthritis, a common complication among patients with a history of a calcaneal fracture. This arthritis is caused by the biomechanical changes of the joint because of the initial trauma, step-off(s), and/or gap(s). Because of these changes, the location of contact pressure in the articular surface differs compared with the anatomical situation, resulting in cartilage destruction, which leads to arthritis.^9^ Thermann et al^20^ stated that the progression of arthritis cannot be predicted in patients with calcaneal fractures. Nonetheless, a large percentage of calcaneal fractures progress to painful posttraumatic subtalar arthritis needing secondary subtalar arthrodesis. In current literature, values up to 29% are mentioned.^3,8,16^

In most cases, an in situ arthrodesis (ISA) is performed. Succinctly, this procedure entails the removal of articular cartilage (débridement) of the subtalar joint, followed by the application of compression through the insertion of 2 or more screws, implemented in various configurations. However, in cases of a substantial loss of calcaneal height, the choice can be made to perform a bone block distraction arthrodesis (BBDA), a technique that was first described by Carr et al. in 1988.^2^ With this technique, a bone block is harvested from a donor site and placed in the subtalar joint (after debridement), resulting in an anatomical restoration of calcaneal height.

The outcome of a secondary subtalar arthrodesis following a previous calcaneal fracture has been widely described. However, the surgical treatment has evolved significantly over the past decade,^18^ paralleling the shifts observed in primary repair strategies. In this study, we describe the outcome following secondary arthrodesis after intra-articular calcaneal fracture, comparing the in situ and bone block distraction techniques, whilst taking into account several possible predictor variables for fusion as well as for the functional outcome and quality of life.

## Methods

### Patient Selection

This study was granted a waiver (22.038) by the Medical Research Ethics Committee of our hospital. A database was created by performing a search in the electronic hospital database for the treatment codes for a pantarsal arthrodesis and an arthrodesis of the subtalar joint. Additionally, a prospective database consisting of patients who underwent surgical treatment for a calcaneal fracture was searched. In total, 339 patients who underwent a subtalar arthrodesis between January 1998 and November 2022 were screened for eligibility. Inclusion criteria were age ≥16 years, having undergone a subtalar arthrodesis following a calcaneal fracture, and a minimal follow-up of 1 year. Exclusion criteria were subtalar arthrodesis before January 2010 and a subtalar arthrodesis within 6 weeks of injury. After a patient was deemed eligible, written informed consent was obtained in accordance with the World Medical Association Declaration of Helsinki.

### Data Collection

The following data were collected: age, gender, body mass index, diabetes, smoking status, ASA score, and referral status. Additionally, fracture and treatment characteristics, surgical approach for arthrodesis, type and amount of screws used for arthrodesis, whether a bone block distraction was performed, the bone block donor site, and incision time were collected. Data were extracted from the electronic patient record.

From 2014 onward, paper surveys were sent to the patients at varying intervals. In December 2022 an equivalent survey was sent using Castor EDC (Castor, Amsterdam, North Holland, the Netherlands) to all patients who had a valid e-mail address, including those who had already received a paper survey. The survey consisted of 3 different questionnaires: the American Orthopaedic Foot & Ankle Society ankle-hindfoot scale (AOFAS),^10^ Foot Function Index (FFI),^1^ and EuroQol-5D-5L (EQ5D-5L).^7^

The AOFAS score represents a survey that specifically addresses hindfoot functionality and stability, with a score extending from 0 (poor function) to 100 (excellent function). Conversely, the FFI evaluates the functionality and stability of the entire foot, with its scoring system inversely ranging from 100 (poor function) to 0 (excellent function). Furthermore, the EQ5D-5L instrument serves as a tool for assessing overall quality of life.

Surgical site infections (SSIs) were scored according to the Centers for Disease Control and Prevention (CDC) criteria.^11^ Subtalar union was scored based on the available radiographs within 1 year after fusion and scored by an experienced foot/ankle surgeon. Fractures that were missed at the initial assessment were scored as being treated conservatively if no ORIF was performed at a later stage.

### Surgical Approach

In 2010, at the beginning of patient inclusion, the extended lateral approach (ELA) and Gallie posterior approach (GPA) were the preferred incisions for performing subtalar arthrodeses in our institute. In this cohort, for ELA, the same steps were followed as described by the paper of Schepers et al,^19^ whereas the technique described by Park et al^15^ was used for GPA.

From 2012 onward, the sinus tarsi approach (STA) became the preferred approach for calcaneal fracture/arthrodesis surgery in our hospital. This shift was made as lower complication rates and a similar outcome were reported, most likely due to the less invasive nature of STA. For STA, the same steps as described previously by Eelsing et al^5^ were followed.

**Figure 1.**
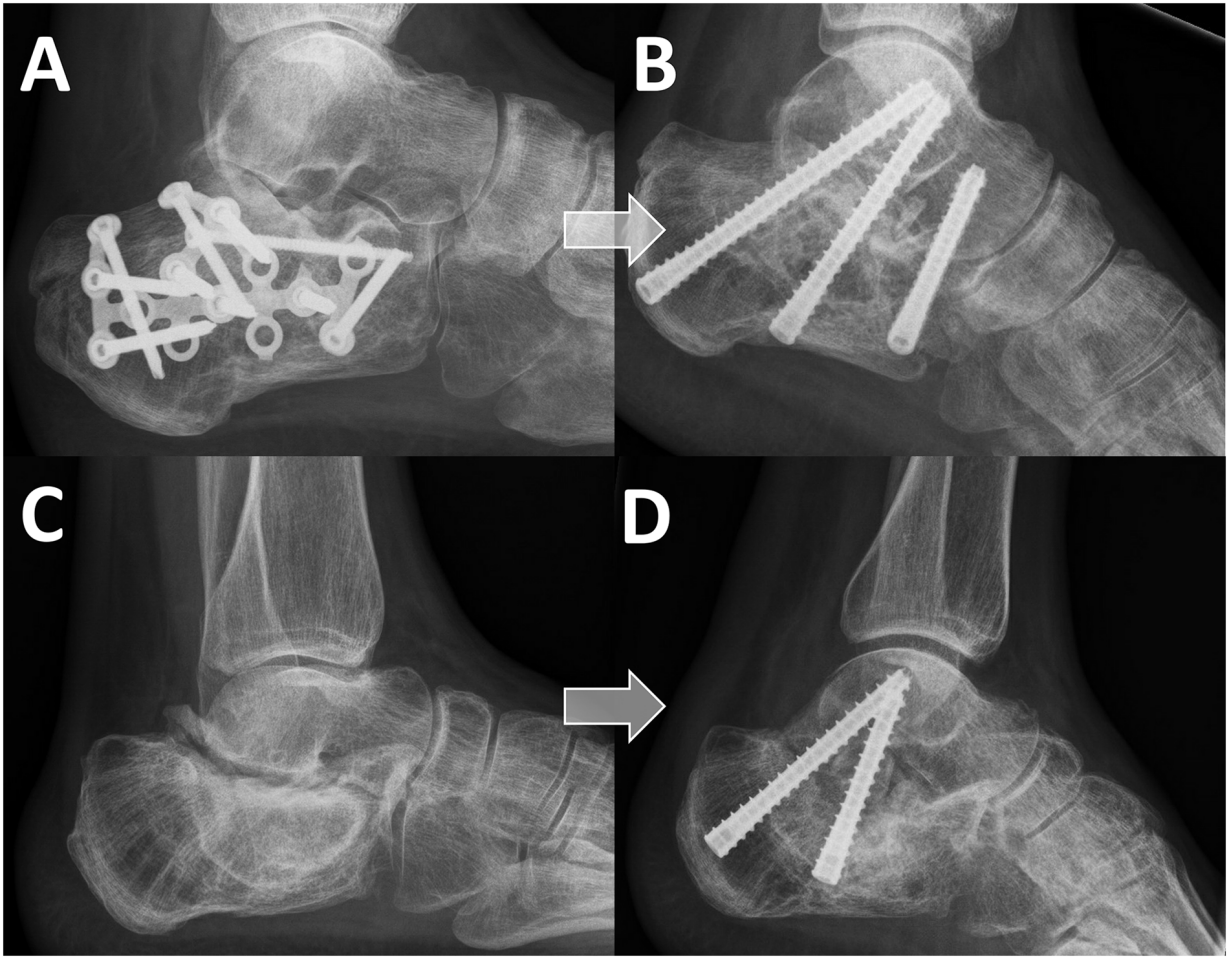
In situ arthrodesis (ISA) and bone block distraction arthrodesis (BBDA) techniques. (A) Before ISA. (B) After ISA. (C) Before BBDA. (D) After BBDA.

The indication whether to perform a subtalar arthrodesis was made by an experienced foot/ankle surgeon with help of the criteria as set out by Schepers^17^: (1) achieving correction of the deformity, (2) relieving pain, (3) stabilizing joints, and (4) improving functional outcome. A BBDA was considered when a reduction in calcaneal height of greater than 5 mm was observed, and specifically performed when there was an 8-mm decrease in height compared to the uninjured contralateral side as depicted on a weightbearing radiograph, based on the indication as described by Myerson and Quill.^13^ Two or more screws implemented in various configurations were used, based on the preference of the surgeon. If arthritis of the talonavicular joint, ankle joint, or calcaneocuboid joint was present, a total ankle arthrodesis or pan-arthrodesis was performed. The ISA and BBDA techniques are illustrated in Figure 1.

### Outcome and Statistical Analysis

The primary outcomes were the differences between ISA and BBDA in surgical outcome as expressed in subtalar union rate, SSIs, IR rate, and need for a revision arthrodesis. Secondary outcomes were the AOFAS, FFI, and EQ5D-5L scores and their possible predictors.

Data were analyzed using the IBM Statistical Package for the Social Sciences (SPSS) version 28.0 (IBM; Armonk, NY, USA). To test for the normality of data, the Shapiro Wilk test was used. When test conditions were met, independent *t* test, χ^2^ test, Mann-Whitney *U* test, linear regression, and Fisher exact test were used. The backward multiple linear regression was performed when 2 or more possible predictor variables (*P* < .1) were identified in the univariate analysis. A 2-sided *P* value of <.05 was considered significant.

## Results

**Figure 2.**
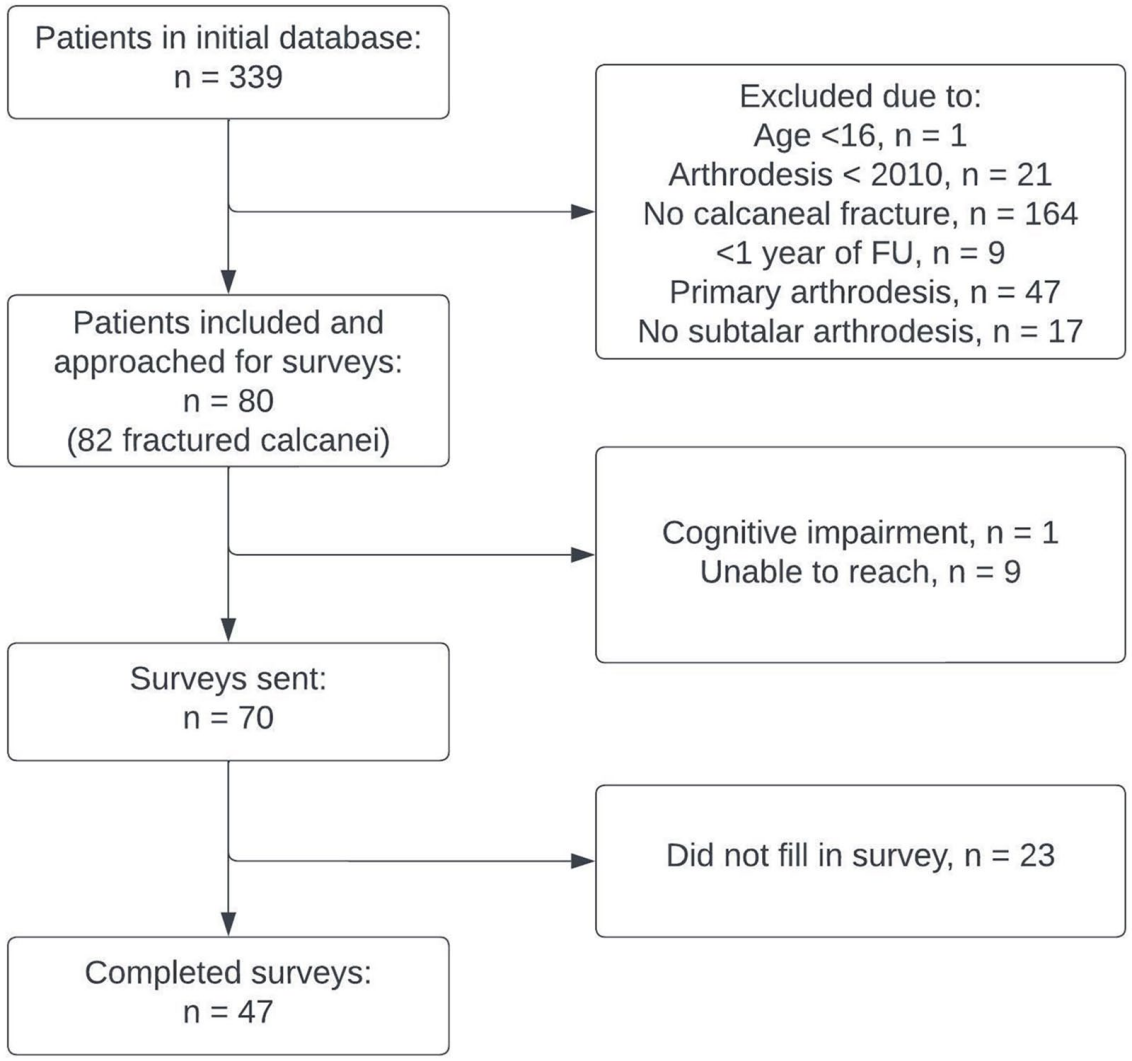
Flow diagram for survey follow-up.

**Figure 3.**
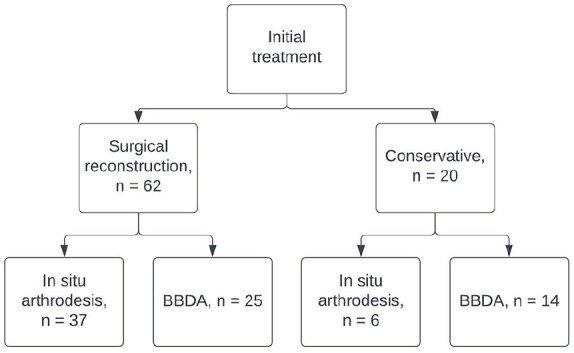
Treatment flow diagram.

Figure 2 shows the flow chart of patient follow-up. Patient characteristics are displayed in Table 1, and fracture and treatment characteristics are shown in Table 2. A total of 259 patients did not meet the inclusion criteria, resulting in the inclusion of 80 patients with 82 fractured calcanei. The median follow-up of the surveys was 51 months (IQR: 25-99), and 49 patients (61%) fully completed the surveys. In the ISA group, 27 patients (63%) completed the survey, compared with 22 patients (56%) in the BBDA group. Figure 3 shows that a BBDA is performed more often after initial conservative treatment (14/20; 70.0%) than after initial ORIF (25/62; 40.3%). Five fractures were missed by the initial assessment and scored as being treated conservatively.

**Table 1.** Patient Characteristics.

Characteristic	Total, n = 80^a^	ISA, n = 43^a^	BBDA, n = 39^a^	*P* Value
Male gender, n (%)	51 (63.7)	27 (62.8)	26 (66.6)	.714
Age, mean (SD)	47.7 (12.8)	47.7 (13.0)	47.0 (12.9)	.808
Body mass index, mean (SD)	25.9 (4.3)	26.1 (4.6)	25.8 (4.0)	.699
Diabetes, n (%)	–	–	–	n/a
Smoking, n (%)	34 (42.5)	18 (41.9)	17 (43.6)	.874
ASA class, n (%)				.881
ASA 1	46 (57.5)	25 (58.1)	23 (59.0)	
ASA 2	31 (38.8)	16 (37.2)	15 (38.5)	
ASA 3	3 (3.8)	2 (4.7)	1 (2.6)	
Referred, n (%)	61 (76.3)	29 (67.4)	33 (84.6)	.071

Abbreviations: ASA, American Society of Anesthesiologists; BBDA, bone block distraction arthrodesis; ISA, in situ arthrodesis.

a Two subjects have a bilateral fracture that was treated with a subtalar arthrodesis; this explains why the sum of the subgroups is not concordant with the total group. One of the subjects received an ISA of the right foot and BBDA of the left foot.

**Table 2.** Fracture and Treatment Characteristics.

Characteristic	Total, n = 82	ISA, n = 43	BBDA, n = 39	*P* Value^a^
Böhler angle at injury, mean (SD)	3.4 (16.0)	4.2 (16.8)	2.1 (14.9)	.652^b^
Open fracture, n (%)	8 (9.8)	3 (7.0)	5 (12.8)	.373^c^
Type of primary treatment, n (%)				**.021** ^c^
Conservative	20 (24.4)	6 (14.0)	14 (35.9)	
ORIF	62 (75.6)	37 (86.0)	25 (64.1)	
Deep SSI after primary treatment, n (%)	19 (23.2)	9 (20.9)	10 (25.6)	.614^c^
Months from injury till arthrodesis, median (IQR)	18.00 (11.0, 33.0)	22.5 (13.8, 42.8)	16 (8.0, 28.0)	**.042** ^d^
Böhler angle before arthrodesis, median (IQR)	13.0 (2.8, 23.0)	20.0 (12.0, 24.0)	3.0 (–12.0, 13.0)	**<.001** ^d^
Indication for arthrodesis, n (%)				**<.001** ^c^
Posttraumatic arthritis	51 (62.2)	35 (81.4)	16 (41.0)	
Malunion/nonunion	31 (37.8)	8 (18.6)	23 (59.0)	
Surgical approach for arthrodesis, n (%)				**<.001** ^c^
GPA	13 (15.9)	3 (7.0)	10 (25.6)	
ELA	36 (43.9)	14 (32.6)	22 (56.4)	
STA	33 (40.2)	26 (60.5)	7 (17.9)	
Type of screws for arthrodesis, n (%)				.122^c^
Acutrak	38 (46.3)	23 (53.5)	15 (38.5)	
Medartis	14 (17.1)	4 (9.3)	10 (25.6)	
Cannulated NFS	30 (36.6)	16 (37.2)	14 (35.9)	
Amount of screws for arthrodesis, n (%)				.381^c^
2	45 (54.9)	26 (60.5)	19 (48.7)	
3	33 (40.2)	16 (37.2)	17 (43.6)	
4	4 (4.9)	1 (2.3)	3 (7.7)	
Bone block distraction donor site, n (%)				n/a
Lateral calcaneal wall	n/a	n/a	26 (66.6)	
Iliac crest	n/a	n/a	10 (25.6)	
Combined lateral calcaneal wall and iliac crest	n/a	n/a	1 (2.6)	
Dwyer	n/a	n/a	1 (2.6)	
Donor femoral head	n/a	n/a	1 (2.6)	
Incision time in minutes, median (IQR)	86 (60-115)	77 (51-102)	95 (77-143)	**.009** ^d^

Abbreviations: BBDA, bone block distraction arthrodesis; ELA, extended lateral approach; GPA, Galli posterior approach; IQR, interquartile range; ISA, in situ arthrodesis; NFS, not further specified; ORIF, open reduction internal fixation; SSI, surgical site infection; STA, sinus tarsi approach.

a *P* values in bold indicate significance (*P* < .05).

b Independent *t* test.

c χ^2^ test.

d Mann-Whitney *U* test.

The surgical and functional outcome are displayed in Table 3. No significant differences between ISA and BBDA in surgical outcome were seen. Subtalar fusion was achieved in 78 of the patients (95.1%). A deep SSI occurred in 6 of the patients (7.8%). Five patients were additionally diagnosed with a deep SSI by cultures subsequent to the initial ORIF, with 4 of these patients presenting an active infection during the arthrodesis procedure. The AOFAS, FFI, and EQ5D index were similar between the 2 groups. However, a significantly higher median EQ5D-VAS was reported by the patients who underwent a BBDA (median [IQR], 70.0 [52.0-82.0] vs 81.0 [70.0-90.3], *P* = .021).

**Table 3.** Surgical and Functional Outcome.

Outcome	Total, n = 82	ISA, n = 43	BBDA, n = 39	*P* Value^a^
Surgical outcome, n (%)				
Subtalar fusion	78 (95.1)	40 (93.0)	38 (97.4)	.617^b^
Deep SSI	6 (7.3)	4 (9.3)	2 (5.1)	.678^b^
Implant removal	10 (12.2)	4 (9.3)	6 (15.4)	.401^c^
Implant removal due to pain	9 (90.0)	3 (7.0)	6 (15.4)	
Implant removal due to infection	1 (10.0)	1 (2.3)	0 (0.0)	
Revision arthrodesis	9 (11.0)	4 (9.3)	5 (12.8)	.611^c^
Functional outcome, median (IQR)				
AOFAS^d^	69.0 (54.5-82.0)	64.0 (54.0-81.0)	71.5 (54.0-82.3)	.488^e^
FFI^d^	30.9 (10.6-58.7)	33.3 (9.7-67.2)	22.71 (11.7-51.2)	.520^e^
EQ5D^d^	0.791 (0.653-0.852)	0.765 (0.609-0.852)	0.802 (0.689-0.861)	.432^e^
EQ5D-VAS^d^	75.0 (62.5-90.0)	70.0 (52.0-82.0)	81.0 (70.0-90.3)	**.021** ^e^
Survey follow-up, median (IQR)	51.0 (25.0-98.5)	51.0 (34.0-94.0)	69.0 (21.0-103.5)	.849^e^

Abbreviations: AOFAS, American Orthopaedic Foot & Ankle Society ankle-hindfoot score; BBDA, bone block distraction arthrodesis; EQ5D, EuroQol–5 Dimensions; EQ5D-VAS, EQ5D visual analog scale; FFI, Foot Function Index; IQR, interquartile range; ISA, in situ arthrodesis; SSI, surgical site infection.

a *P* values in bold indicate significance (*P* < .05).

b Fisher exact test.

c χ^2^ test.

d The survey scores of subjects with a bilateral fracture were included in the group of the foot of which they reported the most complaints.

e Mann-Whitney *U* test.

The results of the univariate analysis can be found in Supplementary Table 1. Of the possible predictors variables, 18 were included in the multiple regression analysis. The results of the multiple regression analysis are shown in Table 4. Multiple regression revealed that a higher Böhler angle prior to the reconstruction significantly improved the AOFAS score, whereas the FFI significantly improved by an initial conservative treatment and implant removal after arthrodesis. Finally, increasing age significantly improved the EQ5D index.

**Table 4.** Multiple regression analysis.

Predictors	*B*	SE	*P* Value
AOFAS			
BA prior to reconstruction	0.384	0.164	.025
FFI			
Conservative	−20.756	9.345	.033
Implant removal after arthrodesis	28.365	13.382	.042
EQ5D			
Age	0.006	0.002	.003
EQ5D-VAS			
n/a	n/a	n/a	n/a

Abbreviations: AOFAS, American Orthopaedic Foot & Ankle Society ankle-hindfoot score; EQ5D, EuroQol–5 Dimensions; EQ5D-VAS, EQ5D visual analog scale; FFI, Foot Function Index.

## Discussion and Conclusion

The results show that, based on our study, the surgical outcome is similar between an in situ arthrodesis and bone block distraction arthrodesis. The functional outcome, as expressed in the AOFAS and FFI surveys, was also similar between both an ISA and BBDA. However, a difference was seen in the reported visual analog scale score of the EQ5D survey in favor of BBDA.

We hypothesized that a possible explanation for this difference could be a difference in the patient’s perspective. As mentioned in the results, patients undergoing a BBDA frequently present as those initially managed conservatively. Consequently, it is conceivable that conservative management may expedite calcaneal collapse and precipitate early-onset traumatic arthritis. Additionally, 75% of the conservatively treated patients were referrals from a local hospital. Given the relatively low incidence of these types of injury, inexperience with the treatment options may therefore play a role. Patients may even disagree with the initial treatment. Subsequently, a phenomenon of patient concordance may arise consequent to the perception of finally receiving surgical intervention and specialized care. This may, hypothetically, explain the higher EQ5D-VAS score despite the absence of disparities in functional outcome and quality of life between the 2 interventions.

As mentioned in the Results section, several predictors for the functional outcome and quality of life were found. They may aid in predicting the outcome after a subtalar arthrodesis with the help of specific patient characteristics. Interestingly, a conservative treatment before the arthrodesis predicts a lower, and therefore better, FFI score. Nonetheless, a better FFI score is not seen in the BBDA group, where 70.0% were treated conservatively before the BBDA compared with 40.3% in the ISA group.

The bulk of academic literature pertaining to subtalar arthrodesis as a treatment modality for subtalar pathology primarily emanates from the period between 1990 and 2010, often characterized by constrained sample sizes.^6^ As delineated in the Introduction section, the surgical treatment for calcaneal fractures has undergone a major shift since 2010. Notably, a prominent shift in surgical technique has been observed, transitioning from the traditional extended lateral approach to the sinus tarsi approach, resulting in a substantial decrease in postoperative wound complications.^4^ This change in surgical approach serves as a primary motivation for the present study. Despite the disparity in postoperative wound complications between ELA and STA, the long-term effects on functional outcome after a displaced intraarticular calcaneal fracture remain unclear.^18^ The current study did not establish the surgical approach as a predictive factor for functional outcomes and quality of life enhancement in the long term after a secondary subtalar arthrodesis.

Patient characteristics did not differ significantly between both the ISA and BBDA groups. However, the type of primary treatment (conservative or operative), months from injury until secondary arthrodesis, Böhler angle before arthrodesis, indication for arthrodesis, surgical approach used for arthrodesis, and incision time differed significantly between both groups. Nonetheless, we posit that, aside from the primary treatment type and Böhler angle, these variables would not exert a substantial influence on the outcomes studied in this cohort. The choice of primary treatment and Böhler angle are directly associated with the indication for employing BBDA as a treatment. Specifically, after conservative management, the likelihood of calcaneal collapse increases because of the absence of internal stabilization as provided by surgical fixation. Additionally, Böhler angle serves as a direct reflection of calcaneal height. Consequently, these potential confounding variables are deemed irrelevant, as they accurately reflect the clinical context in which they would be used to make a treatment decision.

In our opinion, the results of this study can be considered reliable because of the large sample size, simultaneous comparison of the 2 arthrodesis techniques, homogeneity in surgeons performing the surgeries, and length of follow-up. The main limitations are the difference in treatment characteristics between the 2 groups and the unavertable loss to follow-up of 39% of patient-reported outcomes inextricably linked to the retrospective design of the study. Additionally, recent insights suggest that the AOFAS score has fallen out of favor because of its low correlation with other validated outcome measures and it is not a true patient-reported outcome measure—as the clinician must complete part of the score. However, we used another functional outcome measure, the Foot Function Index (FFI), which has a known concern because of a low ceiling effect—which means we probably cannot distinguish between medium- and high-level functioning in our patient group.

A post hoc power analysis was not performed, as the informational value remains debatable.^21^ Additionally, including the needed number of patients would require significant additional effort, funds, and multicenter collaboration given the slim differences in outcome and relatively low incidence of these procedures. To further delineate the observed effects within this investigation, a prospective multicenter trial with a sufficiently powered sample size and rigorous power analysis would be imperative. We suggest to follow the criteria as described by Schepers^17^ in combination with the 8-mm loss of height cutoff value as described by Myerson and Quill^13^ in order to decide when to perform a BBDA.

In conclusion, this article delineates the functional outcome and quality of life following secondary arthrodesis. Our study presents comparable surgical outcomes between in situ arthrodesis and bone block distraction arthrodesis for secondary subtalar arthrodesis following calcaneal fractures. Functional outcomes, as measured by the AOFAS and FFI scores, were also similar between the 2 techniques, although patients undergoing BBDA reported higher EQ5D visual analog scale scores. We further have identified Böhler angle, nonoperative treatment, material removal, and age as predictors for the outcome subsequent to secondary arthrodesis. Even though BBDA is technically more demanding, it appears to not be associated with increased risk and may be worthwhile to reconstruct the height of the calcaneus to obtain similar results as after ISA.

## Supplemental Material

sj-pdf-1-fao-10.1177_24730114241311895 – Supplemental material for Secondary Subtalar Arthrodesis: Surgical Outcome and Predictors of Functional Outcome and Quality of Life After Bone Block Distraction vs In Situ Technique Supplemental material, sj-pdf-1-fao-10.1177_24730114241311895 for Secondary Subtalar Arthrodesis: Surgical Outcome and Predictors of Functional Outcome and Quality of Life After Bone Block Distraction vs In Situ Technique by Robin Eelsing, Sally Al-Sheikh, Jens A. Halm and Tim Schepers in Foot & Ankle Orthopaedics

## Appendix

**Supplementary Table 1.** Univariate Analysis of PROMs.

Variable	AOFAS	FFI	EQ5D	EQ5D-VAS
Age	0.132^a^	**0.094** ^a^	**0.022** ^a^	0.185^a^
ASA	**0.085** ^c^	0.265^c^	**0.023** ^c^	0.170^c^
Body mass index	0.214^a^	0.454^a^	0.136^a^	0.557^a^
Gender	0.856^b^	0.673^b^	0.880^b^	0.881^b^
Smoking	0.277^b^	0.495^b^	0.352^b^	**0.090** ^b^
Referred	0.557^b^	0.792^b^	0.904^b^	0.531^b^
Missed fracture	0.953^b^	0.953^b^	0.485^b^	0.538^b^
BA at presentation	**0.025** ^a^	**0.024** ^a^	**0.031** ^a^	0.816^a^
Open fracture	0.323^b^	0.388^b^	0.284^b^	0.146^b^
Conservative at reconstruction	0.129^b^	**0.019** ^b^	0.122^b^	**0.080** ^b^
Indication for arthrodesis	0.745^b^	0.737 ^b^	0.870 ^b^	**0.091** ^b^
BA before arthrodesis	0.664^a^	0.283^a^	0.552^a^	0.640^a^
Time from primary treatment till arthrodesis	0.203^a^	0.353^a^	0.519^a^	0.326^a^
Surgical approach	0.928^c^	0.866^c^	0.895^c^	0.208^c^
Type of screws	0.634^c^	0.625^c^	0.784^c^	0.483^c^
Amount of screws	0.347^c^	0.313^c^	0.292^c^	0.966^c^
Incision time	0.492^a^	0.825^a^	0.304^a^	0.952^a^
Deep SSI after arthrodesis	**0.016** ^b^	**0.029** ^b^	**0.023** ^b^	0.738^b^
Subtalar fusion	0.303^b^	0.679^b^	0.922^b^	0.485^b^
Implant removal after arthrodesis	**0.011** ^b^	**0.014** ^b^	**0.024** ^b^	**0.091** ^b^
Revision arthrodesis	0.860^b^	0.835^b^	0.324^b^	0.962^b^

Abbreviations: AOFAS, American Orthopaedic Foot & Ankle Society ankle-hindfoot score; ASA, American Society of Anesthesiologists; BA, Bohler angle; EQ5D, EuroQol–5 Dimensions; EQ5D-VAS, EQ5D visual analog scale; FFI, Foot Function Index; PROMs, patient-reported outcome measures. *P*-values in bold indicate a value of <0.1 and were included into the multiple regression analysis.

a = Linear regression.

b = Mann-Whitney *U* test.

c = Kruskal-Wallis test. .
